# 3D-printed gelled electrolytes for electroanalytical applications

**DOI:** 10.1038/s41598-025-90790-x

**Published:** 2025-02-26

**Authors:** Andrzej Krempiński, Konrad Rudnicki, Weronika Korzonek, Lukasz Poltorak

**Affiliations:** 1 Department of Inorganic and Analytical Chemistry, Electroanalysis and Electrochemistry Group, Faculty of Chemistry, University of Lodz, Tamka 12, 91-403 Lodz, Poland; 2 Doctoral School of Exact and Natural Sciences, University of Lodz, Matejki 21/23, 90-237 Lodz, Poland

**Keywords:** Bioprinting, Robocasting, Direct ink writing, Screen printed electrodes, Electroanalysis, Chemistry, Analytical chemistry, Electrochemistry

## Abstract

In this work, several gelators were employed to formulate a conducive gel phase (ionic conductivity) compatible with direct ink writing/bioprinting/robocasting (different names in the literature describe the same printing technology). The main goal of this work was to evaluate gelled phases being a mixture of background electrolyte (NaCl), redox probe (Fe(CN)_6_^3−/4−^), and gel precursor (guar gum, gelatine, agarose, and agar-agar). The studied concentration of gelators ranged from 0.1 to 4% depending on the employed system. Each gelator required a customized formulation protocol. We have found that guar gum exhibits the best printing properties (lack of aggregates blocking the printing nozzle) while giving the least reproducible electrochemical results (when a glassy carbon electrode was employed as the working electrode). The study of two other gelators (agarose and gelatin) indicated significant changes in the electrochemical properties of the investigated surface as their concentration and number of voltammetric scans were varied. The best electrochemical performance was obtained for agar-agar however, this was also a gelator causing the most problems during 3D printing. Finally, we have employed six screen-printed electrodes displaying approximate properties, that were further covered with a 3D-printed conductive gelled cube (direct printing over the electrode surface). We have found that such a system allowed for a surprisingly good electroanalytical response when the model redox probe (Fe(CN)_6_^3−/4−^) was considered. This work is a prelude to 3D-printed gel-based detection devices we are currently developing in our team.

**Supplementary Information:**

The online version contains supplementary material available at 10.1038/s41598-025-90790-x.

## Introduction

Hydrogels are intricate structures formed by interconnected polymer chains that possess a strong affinity to water, allowing them to absorb and retain large quantities of solvent without losing their integrity^1^. The most common substances used for the hydrogel formulation are naturally occurring polymers. In this work, we have focused on four different molecules, this is gelatine, agarose, guar gum, and agar-agar, which are briefly described as follows. Gelatin is a natural polypeptide that is structurally similar to collagen. It is composed of approximately 18 amino acids, with glycine (Gly, about 26%), proline (Pro, about 16%), and 4-hydroxyproline (Hyp, about 16%) being the most abundant. Gelatin does not contain cysteine (Cys) or tryptophan (Trp). The most frequently occurring sequences in the polypeptide is Gly-Pro-X or Gly-X-Hyp, where X is a basic or acidic amino acid. It was found, that gelatin is a suitable modifier of the Au electrode surface as its presence does not provide significant redox interference^2^. Agarose, in turn, is a linear polymer made up of alternating units of d-galactose and 3,6-anhydro-l-galactopyranose, connected by α-(1→3) and β-(1→4) glycosidic bonds. The 3,6-anhydro-l-galactopyranose is an l-galactose molecule with an anhydrous bridge between the 3rd and 6th positions, although some l-galactose units in the polymer may lack this bridge. Some d-galactose and l-galactose units can be methylated, and small amounts of pyruvate and sulfate may be also present. Agar is composed of a blend of two polysaccharides: agarose and agaropectin with the latter being a mixture of d-galactose and l-galactose, which are reach in sulfate, glucuronate, and pyruvate functionalities. Finally, the guar gum is a natural polymer classified as a galactomannan, with d-mannopyranose main chain units linked by β-1,4-glycosidic bonds, and the d-galactopyranose units attached to it via α-1,6-glycosidic bonds.

Both, natural and synthetic hydrogels show great promise for a range of applications. In the realm of energy storage and conversion, hydrogels found applications as electrolytes in capacitors^3,4^) or as hydrogen or oxygen-storing platforms^5,6^). They also excel in the domain of drug delivery^7,8^), offering tunable materials that can be chemically programmed to dislodge gelled frameworks at the point of active substances release, as such offering a matrix for efficient drug transport. In another example, transition metal functionalized hydrogels were used in electrocatalysis^9^ of oxygen evolution reaction (OER). Hydrogels can be used to reduce natural convection and migration in e.g. long-term electrolysis processes. This approach helps to ensure consistent results and aligns well with fundamental theories like the Randles–Sevcik equation, Cottrell equation, and Shoup and Szabo equations, as noted in the indicated studies^10–13^.

Hydrogels play an important role in biosensor development^14–16^), frequently serving as the matrix compatible with the biomolecules (e.g. enzymes) immobilization. In the electrochemistry at the interface between two immiscible electrolyte solutions (ITIES), gelation of one of the phases (usually the organic phase) leads to the improvement of the liquid–liquid interface stability and allows for the analyte interfacial precondensation. Gelled ITIES-based system combined with appropriate electroanalytical techniques e.g. adsorptive stripping voltammetry (AdSV) lowers limits of detection (LODs) and quantification (LOQs). This approach has been used successfully for the determination of substances such as hemoglobin^17^, egg white lysozyme^18^ or insulin^19^. It is obvious that gels are still to be fully discovered in the analytical chemistry field, and one aspect that remains to be defined is controlled gel fabrication, shaping, and their stimuli, induced properties modulation.

Electro-responsive hydrogels can be made out of smart polymers. These materials may change their properties, e.g., shape, and volume, by applying electrical or electrochemical stimuli^20,21^. An example of such materials is thermoresponsive poly(N-isopropylacrylamide) hydrogels modified with dopamine methacrylamide, which exhibit significant shifts in volume phase transition temperature, electroactivity, and responses to catechol oxidation and the presence of Fe^3+^ ions under various pH^22^.

Gel deposits/gelled electrode medium can be pre-formed in a separate reaction vessel (formulation, followed by drop casting and gelation^23,24^)) or via an electrochemically assisted manner. The latter can be controlled with protons (proton-assisted dibenzoyl-l-cystine self-assembly^6,25^) or hydroxyls (fluorenylmethylmethoxycarbonyl polypeptide gelation happening in basic pH range^26^) ions formation during electrochemical water splitting (other reactions changing local pH at the electrode surface can also be applied), or electrochemical formation of the crosslinker (e.g. oxidation of catechol to quinone acting as chitosan crosslinker^27,28^. Printing the gel phase to create functional objects and electrochemical cell counterparts seems to be another intuitive methodology pushing the development of all indicated applications towards the next level. Direct Ink Writing (DIW) is a 3D printing technology that can be applied in this respect. DIW involves the direct extrusion of viscous liquid (ink) in the form of semi-liquid material through the printer nozzle. The ink properties should allow complex structures to be created in a layer-by-layer format, with the printout remaining in its shape at least until the final curing step. DIW is compatible with materials based on hydrogels, synthetic polymers, and in general materials made out of viscous solvents/solutions^29–31^. Frequently, 3D bioprinting, robocasting, and DIW are used alternatively and only the final application governs the name of the used technology^32^.

Bioprinting is used to study tissue regeneration, disease modeling, drug testing, and the creation of organs for transplantation. 3D bioprinted materials can serve as stem cell delivery platforms^33^, or as multicellular structures that can reproduce the architecture and microenvironment of natural tissues^34,35^. Bioprinted tissue models can be used to study mechanisms of cancer development and to test new therapeutic strategies under controlled laboratory conditions^36^. Another application is the development of a ‘liver-on-a-chip’ platform (e.g. to create 3D HepG2/C3A spheroids in GelMA hydrogel^37^) for drug testing, thence offering artificial models to avid animal testing. To further push the development aiming at linking 3D bioprinting with a broad number of the tools provided by electrochemistry we need to understand the link between gel electrochemical properties, their printability, and final platform stability.

In this work, we will discuss the effect of different gelators and their concentrations on the electrochemical properties of model redox couple—hexacyanoferrates, [(Fe(CN)_6_^3−/4−^]—dissolved in the hydrogel formulation. The main goal of this work is to provide printable gels that can be applied for electroanalytical purposes. The effect of the electrochemical parameters and the redox couple concentration on the electrochemical performance of the gel will be discussed. Also, we will prove that direct ink writing can be successfully applied to create a shaped electrolyte medium containing redox active species.

## Methods and materials

### Chemicals

Potassium hexacyanoferrate (III) (K_3_[Fe(CN)_6_], M = 329.26 g⋅mol^− 1^, Chempur, p.a.) and potassium hexacyanoferrate (II) trihydrate (K_4_[Fe(CN)_6_]·3H_2_O, M = 422.41 g⋅mol^− 1^, Chempur, p.a.) were used as a model redox couple dissolved in sodium chloride (NaCl, M = 58.44 g⋅mol^− 1^, Fisher Chemical, ≥ 99.5%) solution. Four gelators were used to prepare the hydrogels: gelatin type B from bovine skin (Sigma-Aldrich), agarose (Serva, research grade), agar-agar (Serva, analytical grade) and guar gum (Sigma–Aldrich). The preparation of each hydrogel is described in the Gelling procedure.

### Electrochemical experiments

All electrochemical experiments were carried out using the AUTOLAB–PGSTAT 204 potentiostat by Metrohm Autolab B.V., The Netherlands. Data analysis and control over applied electroanalytical techniques was conducted using NOVA 2.1 software. Hydrogels electrochemical characterization was performed in a three-electrode configuration with an Ag/AgCl (3 M KCl, BASI®) electrode serving as the reference electrode, a platinum electrode (99.99%, The Min of Poland, Warsaw, Poland) was used as the counter electrode. A glassy carbon electrode (GCE) by BASI®, USA, was used as the working electrode. Before each experiment, the surface of the GCE was polished using aluminium(III) oxide powder by Buehler, USA, first with the particle size of 0.3 μm, and then with 0.05 μm. The mesoporous carbon-modified screen-printed electrodes (SPE) used as the 3D-printed hydrogels supported were purchased from DropSens (model 1100MC).

### Gelling procedure

Guar gum: To prepare the hydrogel, guar gum (0.5%, 1%, 1.5%, 2%, 2.5%, 4% w/w) was added to 15 ml of a x µM solution of [Fe(CN)6]^3−/4−^ dissolved in 250 mM NaCl. Immediately after adding the gelator to the solution, manual stirring was started with a metal spatula at room temperature. The hydrogel was then passed through Acrodisc syringe filter to remove air bubbles. 

Gelatin from bovine skin: To prepare the hydrogel, gelatin (0.5%, 1%, 1.5%, 2%, 4% w/w) was added to 15 mL of a x µM solution of [Fe(CN)6]^3−/4−^ in 250 mM NaCl. The beaker was placed on a magnetic stirrer with a heating plate and the mixture was heated to 40 °C in a water bath. The mixture was stirred until all reagents had dissolved completely and a clear homogenous solution was obtained. 

Agar-agar and Agarose: To prepare the hydrogel, agar-agar or agarose (0.1%, 0.5%, 1%, 1.5%, 2%, 4% w/w) was added to 15 mL of a x µM solution of [Fe(CN)6]^3−/4−^ dissolved in 250 mM NaCl. The beaker was placed on a magnetic stirrer and the mixture was heated to 80 °C in a water bath. The gel was stirred until all reagents had dissolved and a clear homogenous solution was obtained.

### 3D printing

All printouts were made with a Bio X bioprinter by Cellink®. The print-outs were made on a Petri dish or directly on a SPE. Depending on the hydrogel used, different printing parameters such as pressure, and printing speed were used. These parameters were adjusted in situ, during the printing process (printing variables) to ensure the highest level of reproducibility. Some of the parameters were constant for each hydrogel printing process: tool type—pneumatic 3 mL; nozzle diameter—0.410 mm; layer profile—upright square lattice; infill pattern—rectilinear; infill density—100%. Other applied variables include: the printing speed (10 mm·s^− 1^), the back pressure (60 kPa), and the printbed temperature (5 °C).

## Results and discussion

**Fig. 1:**
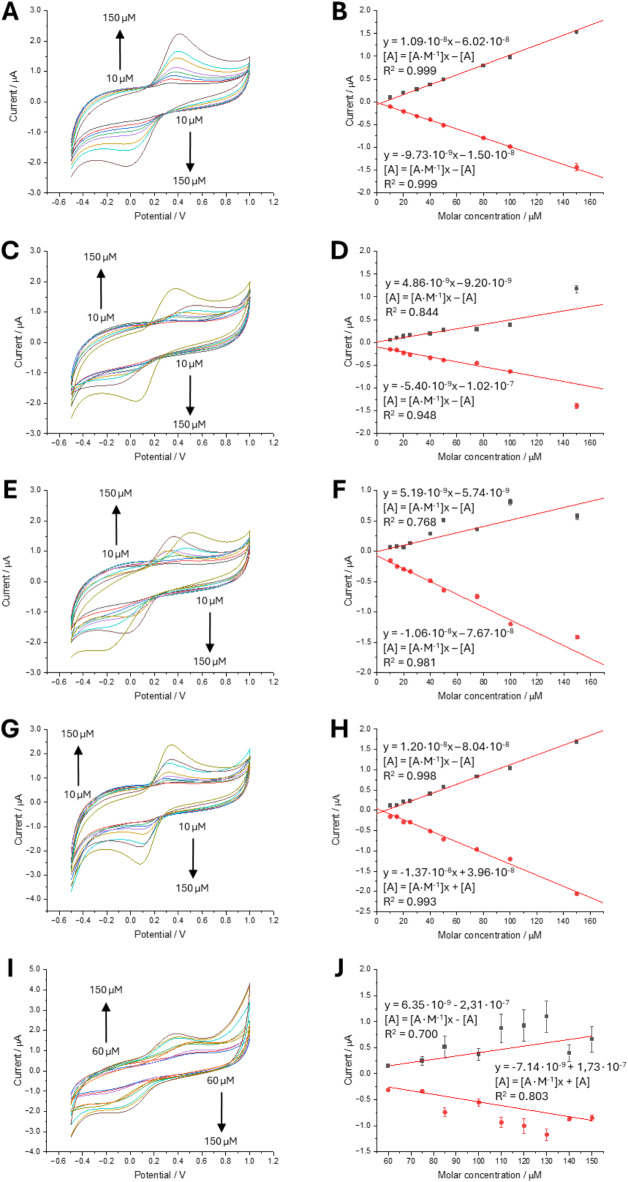
Series of cyclic voltammograms (left panel) and corresponding calibration curves (right panel) recorded in 250 mM NaCl for the increasing concentration of Fe(CN)_6_^3−/4−^ (typically 10, 20, 30, 40, 50, 80, 100, 150 µM; exception I and J—60, 75, 85, 100, 110, 120, 130, 140, 150 µM) gelled with the following gelator: (**A**, **B**)—0% gelator (blank reading); (**C**, **D**)—4% gelatine; (**E**, **F**)—1.25% agarose; (**G**, **H**)—0.5% agar-agar; (**I**, **J**)—4% guar gum. The scan rate was 50 mV·s^− 1^. Each CV was recorded in separate chemical cells. The error bars shown in calibration curves originate from the differences in the intensity of anodic/cathodic currents of the first three voltammetric repetitions. The cyclic voltammograms recorded for other % concentrations of used gelators are available in the electronic supporting information (Fig. S1).

At first, we analyzed the effect of different gelators and their concentrations on the electrochemical properties of [Fe(CN)_6_]^3−/4−^ redox couple (dissolved in the hydrogel formulation before gelator addition and for all [Fe(CN)_6_]^3−/4−^ concentrations) as shown in Fig. 1. [Fe(CN)_6_]^3−/4−^ is considered as a model reversible redox couple with one electron transfer characteristics giving voltammetric features that can be used to evaluate electrochemical properties of the investigated system. The data set from Fig. 1 allowed the inspection of the shape of cyclic voltammograms (CVs), their reproducibility, anodic and cathodic peak intensities, their separation, and any other potential redox features on the combined effect of the redox couple and gelator concentration. Along with the CVs, we show the calibration curves (right panel of Fig. 1) being the dependency of the anodic and cathodic peak currents (averaged over three voltammetric scans, that were 2nd, 3rd, 4th) plotted in function of the [Fe(CN)_6_]^3−/4−^ concentration.

Figure 1A displays the blank reading recorded for the [Fe(CN)_6_]^3−/4−^ in the concentration range from 10 to 150 µM at the GCE placed in the 250 mM NaCl solution. The measured anodic to cathodic peak-to-peak separation (ΔE_p_) was found to increase from 173 mV for 10 µM to 445 mV for 150 µM concentration of the employed redox couple. These values are significantly higher than the theoretical ΔE_p_ value expected for the one-electron reversible system, this is ~ 59 mV at room temperature. Such finding is common for carbon-based electrodes, especially at higher applied scan rate values, and is usually due to slow electron transfer kinetics at the electrode–electrolyte interface. The ratio of the slope of the anodic and cathodic peak current calculated based on the parameters provided in Fig. 1B is equal to 1.1, which indicates that the studied reaction is reversible. Also, as expected, we did not observe any significant variations between voltammetric repetitions which is further confirmed by error bars smaller than the data points (see Fig. 1B) and excellent R^2^ parameters equal to 0.999 for anodic and cathodic signals-based calibration curves. The blank reading along with the extracted voltammetric characteristics will be considered as the reference point.

Next, we started investigating the effect of all employed gelators on the electrochemical characteristic of the [Fe(CN)_6_]^3−/4−^. Figure 1C shows a set of CVs recorded for the 4% gelatine solution. It is important to note that each CV was recorded in a separate cell, and all electrodes were placed into the gelator solution before a sharp rise in viscosity. Electrodes were always occupying the same position and were submerged by a fixed distance (to ensure constant geometrical parameters and spacing between electrodes). The first expected observation is that the position of the anodic and cathodic peak, along with the ΔE_p_ varies as the concentration of the model redox species increases in the range from 10 to 100 µM, reaching the highest ΔE_p_ value equal to 620 mV for 100 µM. The second observation we made, is the two-fold drop of the calibration curve slope (see Fig. 1D) from 1.1∙10^− 8^ and 0.97∙10^− 8^ to 0.49∙10^− 8^ and − 0.54∙10^− 8^ (A∙M^− 1^), for anodic and cathodic currents, respectively, in the absence and the presence of 4% gelatin in the aqueous phase. This is an expected observation, as along with the increasing viscosity diffusion coefficients should drop, thence giving lower resulting Faradaic currents. A gelled phase framework is frequently built from a chemical structural unit bearing charged functionalities that may interact with other molecules via specific and non-specific interactions (e.g. formation of hydrogen bonding, electrostatic interactions) which also affect the diffusivity which is described by the so-called apparent diffusion coefficient. As shown by Howells et al.. the interaction between the silica gel (with the pore walls holding a net negative charge) and positively charged redox probes such as cobalt (II) tris(bipyridiny) and ferrocenylmethyltrimethylammonium iodide resulted in a gradual decrease in the apparent diffusion coefficient spanning over 30 days leading to a conclusion that the specific interaction between silica gel pore walls and the entrapped redox probes is the key factor affecting resulting electrochemical response^38^. For gelatin, the reproducibility of the current-potential curves was average as the calibration curves from Fig. 1D provided correlation coefficients equal to 0.844 and 0.948 for the anodic and cathodic signals, respectively. All this suggests that the interactions between [Fe(CN)_6_]^3−/4−^ and gelatin chains vary between experiments. The pH of the aqueous phase was around 6.0 meaning that amine groups of the lysine subunits will be positively charged. This indicates that electrostatic interaction between the positively charged gelator framework and negatively charged redox probe may affect the final electrochemical response.

The following gelator used to affect the viscosity of the aqueous phase electrolyte was agarose with the resulting set of CVs shown in Fig. 1E and corresponding calibration curves displayed in Fig. 1F. Here, we have observed significant fluctuations in the anodic to cathodic peak to peak separation that reached 750 mV for the highest studied [Fe(CN)_6_]^3−/4−^ concentration equal to 150 µM. The anodic and cathodic-based calibration curves displaced low coefficients of determinations that were 0.768 and 0.981 (for the anodic and cathodic currents, respectively) indicating low reproducibility of the electrochemical data. The slope of the calibration curve plotted based on the anodic signal was equal to 5.19∙10^− 9^ A∙M^− 1^ and was two times lower than the slope of the calibration curve obtained for the non-gelled solution. Also, we have found that the anodic signals were on average two times lower in intensity as compared to the reduction peaks. These observations suggest that (i) it is the oxidized form, this is [Fe(CN)_6_]^3−^, pre-concentrated at the electrode surface after anodic voltammetric sweep, rather than (ii) the rise in the solution viscosity, as we would expect to observe similar current value reduction for the cathodic signals.

The last two studied hydrogels were agar-agar and guar gum. Figure 1G shows a set of CVs along with the corresponding calibration curves recorded for agar-agar gelled electrolytes. From the electroanalytical point of view, this gelator provided the best reproducibility as we did not observe significant fluctuation in the anodic and cathodic peak currents, with the lowest peak-to-peak separation value equal to 200 mV for 150 µM of [Fe(CN)_6_]^3−^, high reproducibility of the recorded voltammograms and calibration curves with a coefficient of determination 0.998 and 0.993 (for anodic and cathodic signals, respectively) reaching satisfactory levels.

We have also found, that in the presence of agar-agar, the voltammetric detection sensitivity towards the employed redox probe increased by around 10% (for the anodic signals) and 30% (for the cathodic signals) as compared to non-gelled system. The difference between agar-agar and agarose is the presence of agaropectin in the formulation of the first gel precursor formulation. Agaropectin is a sulfated galactose-based polysaccharide that is expected to govern the net negative charge of the gel framework. As such, we suspect that the negatively charged probe present at the GCE surface will suppress the diffusion of the redox probe towards the bulk phase, and a surface pre-concentration effect may occur. The last studied gelator was guar gum with the resulting electrochemical output shown in Fig. 1I and J (CVs and calibrations curves, respectively). Here, we have encountered significant changes in the shape of the voltammetric curves as the number of recorded cycles increased (increasing peak-to-peak separation and dropping anodic and cathodic currents). The latter is especially visible in Fig. 1J as the large error bars were obtained especially for the higher concentration of the used redox probe. For all studied cases, lower concertation of the gel precursors provided CVs with higher Faradic currents, still burdened with poor reproducibility (see Fig. S1 from electronic supporting information).

**Fig. 2:**
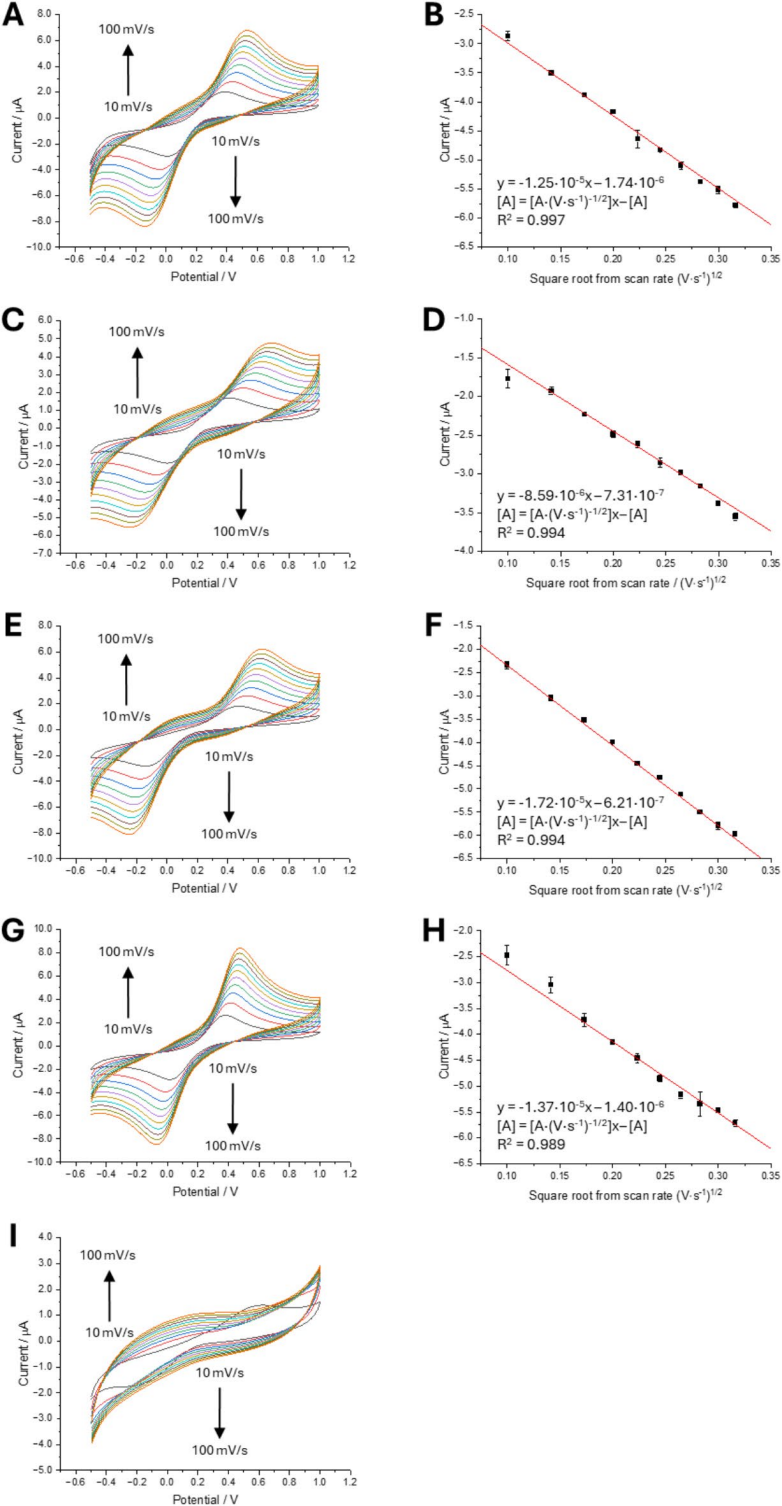
CVs (left panel) along with the corresponding current versus square root from scan rate dependency (right panel) recorded for fixed concentration of Fe(CN)_6_^3−/4−^ equal to 500 µM in 250 mM NaCl in the gelled electrolyte: (**A**, **B**)—0% gelator (blank reading); (**C**, **D**)—4% gelatine; (**E**, **F**)—1.25% agarose; (**G**, **H**)—0.5% agar-agar; (**I**)—4% guar gum. Scan rate values were in the range from 10 to 100 mV·s^− 1^ with 10 mV·s^− 1^ intervals. The current versus square root of scan rate dependency is missing for I due to the high impact of the guar gum on the electrode electrochemical properties (surface passivation). The error bars in the right panel (current vs square root from scan rate dependency) are calculated as the average of the last three (out of four) faradaic currents from multiple voltammetric scans.

According to the Randles–Sevcik equation, voltammetry gives direct access to the assessment of the diffusion coefficient (D) of the redox probes. Figure 2A–I show a set of CVs recorded for the increasing scan rate values (in the range from 10 to 100 mV∙s^− 1^ every 10 mV∙s^− 1^) along with the cathodic peak current intensities plotted in function of the square root from the scan rate dependencies. Using the Randles–Sevcik equation which is shown below:

1 $$\:{\text{I}}_{\text{p}}=2.69\cdot\:1{0}^{5}\cdot\:{\text{n}}^{\frac{3}{2}}\cdot\:\text{A}\cdot\:{\text{D}}^{\frac{1}{2}}\cdot\:{\text{v}}^{\frac{1}{2}}\cdot\:\text{C}$$

where I_p_ is the net peak current (Amps); n is the number of electrons exchanged in the studied redox reaction (1); A is the electrode area (cm^2^); D is the (apparent) diffusion coefficient (cm^2^·s^− 1^); C is the bulk redox concentration (mol·cm^− 3^); and v is the scan rate (V·s^− 1^), we can estimate the value of D once all other parameters are known. Proper rearrangement of the Eq. 1 allows us to correlate the slope of the current plotted in function of the $\:{\text{v}}^{\frac{1}{2}}$ with D, which was calculated for different gelators as shown in Table 1.

In general, the D values in gelled electrolytes (i) were lower than the literature and calculated values for the non-gelled solutions; and (ii) were dropping as the concentration of gelator was increased. These observations are due to the hindered permeability of the analyte in the gelled phase, which is postulated to be caused by steric hindrance and interactions between the redox probe and the polymeric network forming the gel scaffold. Initially, we assumed that for each studied scenario the D would be significantly lower than 7.60·10^− 6^ cm^2^·s^− 1^, however at 0.25% and 0.50% agarose, the D values were found to be 7.57·10^− 6^ cm^2^·s^− 1^ and 6.51·10^− 6^ cm^2^·s^− 1^, respectively. This indicates that either (i) the apparent concentration of the redox probe at the GCE surface is higher than the bulk concentration or (ii) the polymeric framework making the hydrogel scaffold does not form a steric obstacle to the employed redox probe. We think that the latter explanation is not probable as to induce porosity in the hydrogel framework one would require to utilize sophisticated approaches which was not the case for the present work^39^. Nevertheless, for most of the studied gel-precursors-based formulations, we have observed a drop in the redox probe diffusivity. This is, the diffusion coefficient values for 1.25% agarose and 0.25% agar-agar are around two times lower, while for 0.50% agar-agar four times lower as compared to the reference value obtained for the non-gelled solution. In the case of 2–4% gelatine and 0.5% agar-agar, the diffusion coefficients decreased significantly indicating high-gelled framework compactness. We also plotted log(I) versus log(v), as shown in Fig. S3, and found that the slope of the resulting linear dependency consistently fell below 0.5, which is typically expected for diffusion-controlled reversible reactions. The observed slopes ranged from 0.30 to 0.42, indicating that other kinetic factors must be considered. These low values suggest that the current is likely influenced by the insufficient availability of the redox probe species, which may not reach the electrode surface within the time frame of a single voltammetric experiment due to elevated viscosity. 

**Table 1:** Calculated diffusion coefficients for non-gelled Fe(CN)_6_^3−/4−^ solution and gelled solutions with different gelator concentrations.

Type and concentration of gelling agent	D [cm^2^·s^− 1^]	Error*
0.1 M KCl	7.60·10^− 6^	–
4.00% gelatine	8.20·10^− 7^	3.9%
2.00% gelatine	1.18·10^− 6^	4.2%
1.25% agarose	3.29·10^− 6^	1.2%
0.50% agarose	6.51·10^− 6^	2.9%
0.25% agarose	7.57·10^− 6^	3.6%
0.50% agar-agar	2.08·10^− 6^	3.3%
0.25% agar-agar	3.25·10^− 6^	1.7%

The first value in the table is taken from the literature^40^. Displayed data points are taken from the current versus square root from scan rate dependencies shown in Fig. 2 and Fig. S2 from ESI. The error given in the table is the standard error of the slope of the dependency between the current intensity and the square root of the scan rate that was used to calculate the diffusion coefficients.

**Fig. 3:**
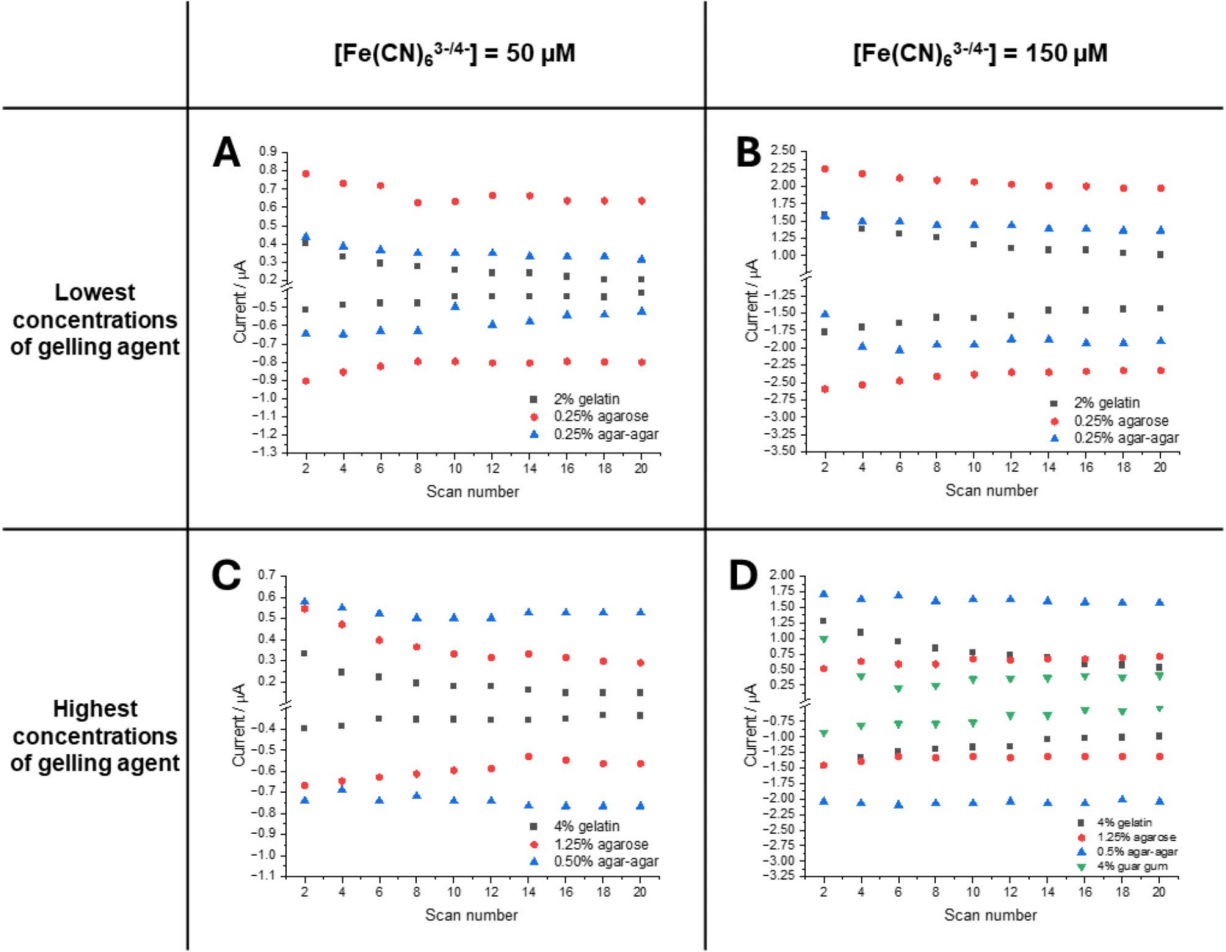
Series of anodic and cathodic peak currents plotted versus voltammetric scan number plots recorded for a fixed concentration of Fe(CN)_6_^3−/4−^ equal to 50 µM (**A**, **C**) and 150 µM (**B**, **D**) in 250 mM NaCl in the gelled electrolyte: (**A**, **B**)—the lowest of the selected gelator concentrations; (**C**, **D**)—the highest of selected gelator concentrations. Type of a gelator is indicated in the figure legend. Displayed data points are taken from the CVs shown in the ESI as Fig. S4.

In all studied cases, the faradaic currents associated with the Fe(CN)₆^3−/4−^ redox reaction decreased following the application of a gelled electrolyte. As previously discussed, this decrease is linked to the declining apparent diffusion coefficients. Another critical factor affecting the efficiency of the electroanalytical signal is the stability of the faradaic current throughout the voltammetric cycling, as investigated and illustrated in Fig. 3. For all systems employed, the absolute values of the measured currents declined after 20 cycles, with the greatest drop observed for gelatin and agarose. For example, the current decrease for 2% gelatin and 50 µM Fe(CN)₆^3−/4−^ was 50%, while for 0.25% agarose and the same concentration of redox probe, the decrease was 19%, as depicted for the anodic signals in Fig. 3A. For these two gel precursors, similar trends were observed across all experimental conditions, including variations in gel precursor concentration (Fig. 3, columns) and redox probe concentration (Fig. 3, rows). 

A particularly noteworthy set of data was obtained for agar-agar, especially at higher gelator concentrations of 0.50%. Here, a stable peak current was observed (Fig. 3D), and there was a slight increase (a few percent) in both cathodic and anodic currents after the last recorded cycle. Additionally, the current remained stable despite the increase in electrolyte viscosity. For guar gum, only data from the highest studied gel concentration (4%) and redox probe concentration (150 µM) could be analyzed. The cyclic voltammograms (CVs) recorded under other experimental conditions displayed excessive resistivity, hindering proper data treatment. A significant drop in the anodic current, along with increasing separation between the anodic and cathodic peaks (as discussed below), indicates a strong interaction between the guar gum gel precursor and the electrode surface.

**Fig. 4:**
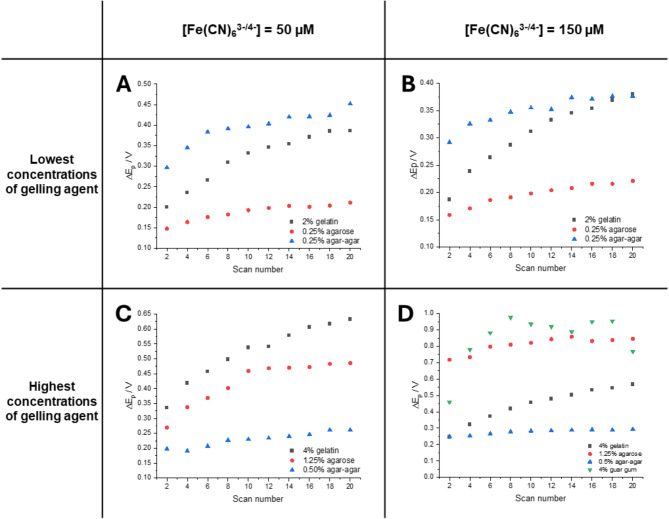
Series of ΔE_p_ (the difference between the anodic and cathodic peak potentials) plotted versus voltammetric scan number recorded for fixed concentration of Fe(CN)_6_^3−/4−^ equal to 50 µM (**A**, **C**) and 150 µM (**B**, **D**) in 250 mM NaCl in the gelled electrolyte: (**A**, **B**)—the lowest and (**C**, **D**)—the highest of studied gelator concentrations. The type of a gelator is indicated in the figure legend. Displayed data points are taken from the CVs shown in the ESI available as Fig. S4.

Figure 4 presents a series of ΔE_p_ (the difference between the anodic and cathodic peak potentials) obtained from CVs recorded for a fixed concentration of Fe(CN)_6_^3−/4−^ of 50 µM (Fig. 4A—lowest studied gelator concentration; Fig. 4C—highest studied gelator concentration) and 150 µM (Fig. 4B—lowest studied gelator concentration; Fig. 4D—highest studied gelator concentration), all dissolved in 250 mM NaCl in the gelled electrolyte. The change in ΔE_p_ was analyzed as a function of the number of consecutively recorded voltammetric scans. Generally, physicochemical changes in the properties of the electrode surface or the adjacent electrolyte (either blocking or catalytic) provide insights into potential surface passivation and/or adsorption/deposition processes, as well as physical alterations in the electrochemical surface area and the electric double layer. As indicated in Fig. 4, for all experimental conditions studied, the peak-to-peak separation increased with the number of applied voltammetric cycles. This trend suggests an increase in charge transfer resistance during the redox reactions, likely due to the electrochemically induced formation of a gel precursor-based film on the electrode surface. Notably, electrodes left in the gel solution at open circuit potential did not exhibit this dependency (data not shown). Figure 4A shows a non-linear increase in anodic and cathodic peak-to-peak separation for all gelators studied. The largest increase was observed for the 2% gelatin solution, with a total increase of 0.186 V (denoted as δΔE_p_), calculated as the difference between the first and last scan (20th cycle). Gelatin, a polypeptide commonly used to modify carbon-based materials like carbon nanotubes, facilitates adsorption through interactions between the hydrophobic domains of the amino acid building blocks and the hydrophobic carbon surface^41^. Since gelatin functions as a polyelectrolyte, its net charge is influenced by solution pH, ionic strength, and the type of gelator used (either type A or type B). Polarization of the electrode surface can enhance the adsorption process through electrostatic attraction or even through the electrochemical induction of the polyelectrolyte charge. This effect is further illustrated in Fig. 4B, C, and D, with the most significant observable effect recorded for the 4% gelatin and 150 µM Fe(CN)_6_^3−/4−^, which yielded a δΔE_p_ of 0.321 V (see Fig. 4D). 

Interestingly, when agar-agar was used as the gel precursor, we observed δΔE_p_ variations of 150 mV and 75 mV for 0.25% agar-agar with 50 µM and 150 µM Fe(CN)_6_^3−/4−^ concentrations, respectively. Increasing the agar-agar concentration to 0.5% yielded a beneficial effect, as ΔE_p_ for the first scan decreased by approximately 100 mV for the first cycle, and δΔE_p_ dropped to a negligible 10–20 mV (see Fig. 4C and D). This suggests that agar-agar, which possesses a net negative charge due to the presence of agaropectin, does not block the surface of the glassy carbon electrode (GCE) and may enhance its properties by allowing for the preconcentration of the redox probe at the electrode surface. The negatively charged Fe(CN)_6_^3−/4−^ is likely hindered from diffusing away into the bulk phase due to the negative charge of the gel framework. 

In contrast, the δΔE_p_ for agarose was found to be higher, measuring 62 mV for 0.25% agarose with 50 µM Fe(CN)_6_^3−/4−^, 70 mV for 0.25% agarose with 150 µM Fe(CN)_6_^3−/4−^, 200 mV for 1.25% agarose with 50 µM Fe(CN)_6_^3−/4−^, and 100 mV for 1.25% agarose with 150 µM Fe(CN)_6_^3−/4−^. This data indicates that agaropectin not only enhances the electroanalytical performance of the system but also prevents the electrode surface from becoming blocked, a situation that arose when agarose was used as a gel precursor. These findings indicate that agar-agar enhances the electroanalytical performance of the developed platform, while agarose and gelatin likely result in some degree of passivation of the electrode surface. Despite this passivation, both agarose and gelatin still allow for access to valuable electrochemical information regarding the redox probe. In contrast, guar gum presented the most significant challenges, as it strongly influences the electroanalytical readout, which is reflected in rapid changes in the shape of the cyclic voltammograms (CVs).

Finally, we utilized all formulated hydrogels as inks for the 3D printing (Direct Ink Writing, DIW) of a simple shape - a cube with dimensions of 10 mm × 10 mm × 1 mm. The objective was to assess the printability of the gels and evaluate the durability of the printed objects over time. Gelator concentrations ranged from 0.1 to 4%. The resulting gels were classified into three categories: (i) semi-liquid, (ii) a rigid structure that releases water over time, and (iii) a rigid structure that maintains its shape for at least 24 h. The stability of the gel is essential to maintain the shape of the print out after ink extrusion. Consequently, a concentration of 0.1% (falling into category i) was deemed unsuitable for each gelator. For gelatin, concentrations of 2% and 4% resulted in the formation of stable structures that met the criteria for category (iii). However, both concentrations exhibited issues with nozzle obstruction during the printing process, even when the maximum pressure of 200 kPa was applied. The resulting print outs were incomplete, indicating that gelatin was unsuitable for this study. Agarose and agar-agar, at concentrations of 0.25% and 0.5%, respectively, formed stable structures that released water over time (category ii). Consequently, higher concentrations ranging from 1 to 1.5% were tested as printing inks. Although increasing the concentration of the gel precursor enhanced its rigidity, we encountered frequent nozzle clogging during the printing process, even at the maximum applied pressure of 200 kPa. Ultimately, we focused on guar gum, testing two different concentrations: 2.5% and 4%. At the 2.5% concentration, the guar gum initially formed a compact structure but subsequently lost its shape (category ii/iii). In contrast, the 4% guar gum concentration yielded stable printouts that could be extruded consistently over the printing support, demonstrating high repeatability and an absence of printing artifacts.

**Fig. 5:**
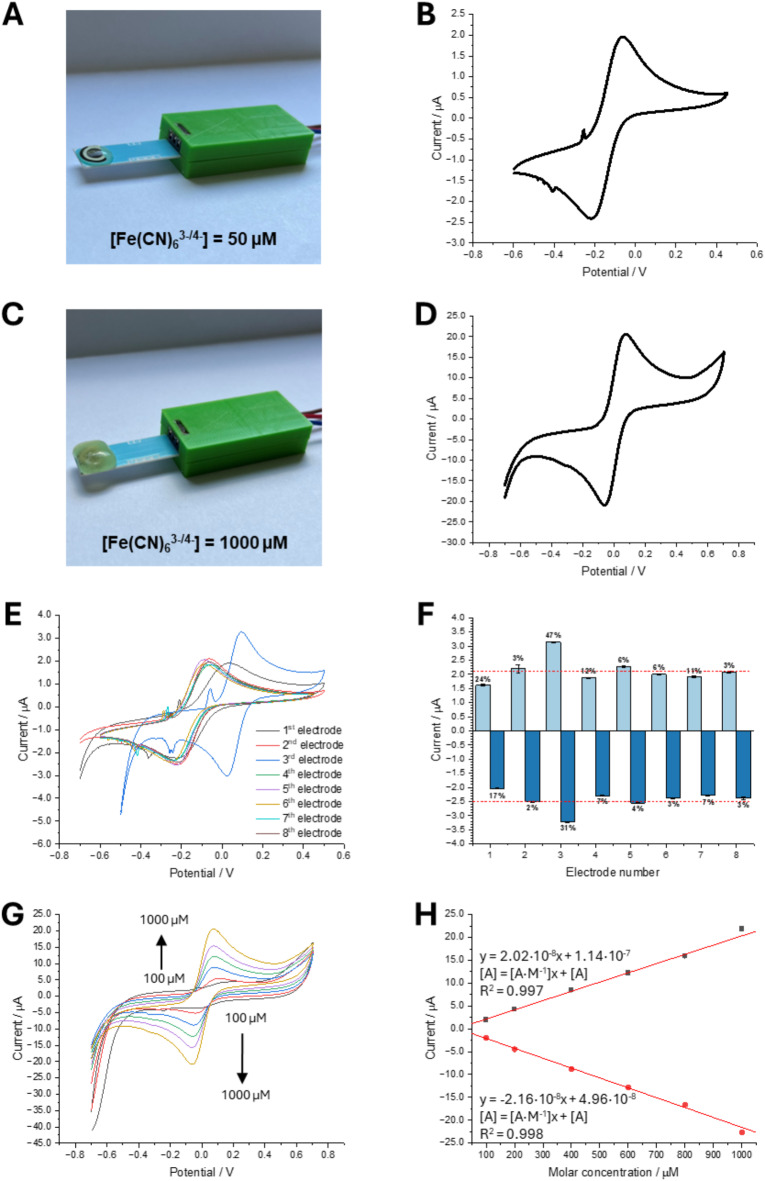
(**A**)—Electrochemical system based on SPE (screen-printed electrode) with a drop of a solution of Fe(CN)_6_^3−/4−^. (**B**)—CV recorded for fixed Fe(CN)_6_^3−/4−^ concentration equal to 50 µM in 250 mM NaCl. (**C**)—Electrochemical system based on SPE with 3D printed 4% guar gum-based hydrogel cube. (**D**)—CV recorded in 4% guar gum-based hydrogel for a fixed concentration of Fe(CN)_6_^3−/4−^ equal to 1000 µM in 250 mM NaCl. (**E**)—CV recorded for the fixed concentration of Fe(CN)_6_^3−/4−^ equal to 50 µM in 250 mM NaCl on eight different SPEs (non-gelled solution). (**F**)—Current versus electrode number plot recorded for fixed concentration of Fe(CN)_6_^3−/4−^ equal to 50 µM in 250 mM NaCl. Error bars are obtained based on three independent measurements. The red dotted lines show the averaged anodic (2.10 µA) and cathodic current (-2.50 µA) calculated for eight electrodes. Percentage numbers placed next to bars indicate deviation from the averaged values. (**G**)—CVs recorded in 4% guar gum-based hydrogel 3D printed over SPE for increasing concentration of Fe(CN)_6_^3−/4−^ along with the calibration curves shown in panel H.

In the end, we have employed the most demanding (from the electrochemical point of view) gel, which was based on guar gum, to print the rigid electrolyte over the surface of screen-printed electrodes (SPE). The guar gum-based printout had the shape of a cube entirely covering all three electrodes (working, counter, and reference electrode). Guar gum was chosen as it gave the most rigid printable shapes among all studied gel precursors (for the investigated concentrations). Figure 5A and B show the image of the SPE with a droplet being the solution of 50 µM Fe(CN)_6_^3−/4−^ dissolved in 250 mM NaCl along with the corresponding CV, respectively. The resulting current potential dependency holds the expected shape with anodic-to-cathodic peak-to-peak potential separation equal to around 150 mV. Current spikes occurring < − 0.2 V are most probably redox-active electrode surface contaminations. Next, we placed the SPE electrode on a printer plate and extruded the hydrogel using a nozzle with 0.41 mm, rectilinear infill pattern, 100% infill density, upright square lattice as layer profile using pneumatic mode, and a syringe having a volume of 3 mL filled with the gelled electrolyte. Other applied variables include: the printing speed (10 mm·s^−1^), the back pressure (60 kPa), and the printbed temperature (5 °C). The printed gel-based cube is shown in Fig. 5C. As already observed for the macroscopic system based on the GCE and by many others, the increased viscosity of the gelled phases results in the dropping values of the diffusion coefficients which is directly observed as the lower magnitudes of the faradaic currents. Due to lower apparent diffusion coefficients of Fe(CN)_6_^3−/4−^ in the gelled phase we have increased the concentration of the redox probe in the gelled matrix to 1000 µM and recorded the CV shown in Fig. 5D. Interestingly, the additional current spikes recorded previously disappeared, and well-shaped CV was obtained with the peak to peak separation further lowered to 140 mV. Subsequently, eight SPE electrodes were examined by drop casting a solution of 50 µM Fe(CN)_6_^3−/4−^ in 250 mM NaCl solution onto the surface of each followed by recording four CV scans—see Fig. 5E. The average anodic and cathodic current was then calculated (based on three repetitions) and plotted in function of the applied electrode number giving a set of data shown in Fig. 5F. Electrodes with a cathodic current error exceeding 10% of the averaged value (− 2.5 µA) were excluded from further analysis, this is electrode number 1 (17% of a difference) and electrode number 3 (31% of a difference). These two electrodes were also giving signals deviating from the employed series as electrode number 1 gave higher charge transfer resistance (increased anodic to cathodic peak-to-peak separation), whereas for electrode number 3 we observe the shift in the half-wave potential most probably due to pseudo reference electrode properties. At the surface of the remaining six SPE electrodes, we have 3D printed a hydrogel-based cube (each cube represented an individual experiment) being a solution of Fe(CN)_6_^3−/4−^ at a concentration from 100 to 1000 µM. Figure 5G is the overlay of six CVs recorded at six different electrodes. Indicated data set shows that the half-wave potential is slightly shifted towards the anodic potential (as compared with the non-gelled electrolyte) which may have to do with the gel affecting the potential of the pseudo-reference electrode. Nevertheless, although six different electrodes were used, the shape of CVs and the position of the anodic and cathodic signals can be considered stable. Also, we did not encounter any other additional current spikes/peaks indicating high stability and reproducibility of the created platform. This is further confirmed in Fig. 5H where we show the calibration curves plotted based on Fig. 5G analysis. High R^2^ values (0.997 and 0.998 for anodic and cathodic signals, respectively) and similar slopes for both redox reactions (2.02·10^− 8^ and − 2.16·10^− 8^ A∙M^-1^) are observed and indicate redox reaction reversibility in the studied 3D printed matrix.

This initial set of data shows that the electrified solid interfaces contacted with the hydrogel-based electrolyte require individual characterization prior to use. The interaction between the hydrogel framework (electrostatic, non-specific) and redox probes (or eventually redox active analyte) is expected to govern the final electroanalytical output. The idea behind this work was to find a printable hydrogel that can be used for potential sensing unit development that can be fabricated using 3D printing technology. In the future, we want to 3D print a self-sustainable gel-based sensing unit that can be applied for analyte pre-concentration and detection.

## Conclusions

In this work, we have investigated the electrochemical properties of four hydrogel precursors (gelatine, agarose, agar-agar, and guar gum) using a three-electrode system and cyclic voltammetry. Studied experimental variables include (i) type of the gel precursor, (ii) its concentration (0.25 − 4% w/w—hydrogel precursor dependent) (ii) the concentration of the model redox couple (Fe(CN)_6_^3−/4−^, typically from 10 to 150 µM, only for guar gum from 60 to 150 µM); and (iv) hydrogel-based electrolyte contacting with the surface of electrode either based on the glassy carbon electrode entrapment in the hydrogel framework or direct hydrogel deposition via ink writing 3D printing technique. The best electrochemical response of the Fe(CN)_6_^3−/4−^ was observed for agar-agar and gelatine (high R^2^ values, well-formed voltammograms, low variation in the electrochemical features measured in function of voltammetric cycling). The second evaluated aspect was the gel printability. The best gelling agent providing superior printing properties was guar gum, which could be used to print cubes with the most stable shape and lack of printing defects. The guar gum-based gel cubes were subsequently printed over a carbon-based screen-printed electrodes surface and were tested electrochemically. We have obtained a response showing a linear increase in the anodic and cathodic currents attributed to Fe(CN)_6_^3−/4−^ redox reactions giving R^2^ of 0.997 and 0.998 in the concentration range from 100 to 1000 µM, respectively. This initial set of data shows that printable gels can be used for electrochemical applications, and in particular electroanalytical studies. In the future, we plan to use extrusion-based techniques to 3D print self-sustaining gelled sensors.

## Electronic supplementary material

Below is the link to the electronic supplementary material.

Supplementary Material 1

## Data Availability

The datasets generated and/or analysed during the current study are available in the ZENODO repository, 10.5281/zenodo.13790175.
