# Supplementary material for: 3D-printed gelled electrolytes for electroanalytical applications
Source: Sci Rep. 2025 Feb 26;15:6917. doi: 10.1038/s41598-025-90790-x (PMC11865509; doi:10.1038/s41598-025-90790-x)
Supplement: Supplementary file 1 — Supplementary Material 1 [file 41598_2025_90790_MOESM1_ESM.docx]

**ELECTRONIC SUPPORTING INFORMATION**

**3D-printed gelled electrolytes for electroanalytical applications**

Andrzej Krempiński,^a, b^ Konrad Rudnicki,^a*^ Weronika Korzonek,^a^ Lukasz Poltorak^a**^

1. Department of Inorganic and Analytical Chemistry, Electroanalysis and Electrochemistry Group, Faculty of Chemistry, University of Lodz, Tamka 12, 91-403 Lodz, Poland
2. Doctoral School of Exact and Natural Sciences, University of Lodz, Matejki 21/23, 90-237 Lodz, Poland

*Corresponding author: [konrad.rudnicki@chemia.uni.lodz.pl](mailto:konrad.rudnicki@chemia.uni.lodz.pl)

**Corresponding author: [lukasz.poltorak@chemia.uni.lodz.pl](mailto:lukasz.poltorak@chemia.uni.lodz.pl)

**Table of contents:**

1. Cyclic Voltammograms – effect of gel precursors concentration – page 2
2. Current vs square root from scan rate dependencies – page 3
3. Log(I) vs log(v) dependencies – page 4
4. Cylic Voltammgorams – effect of consecutive cycling – page 5


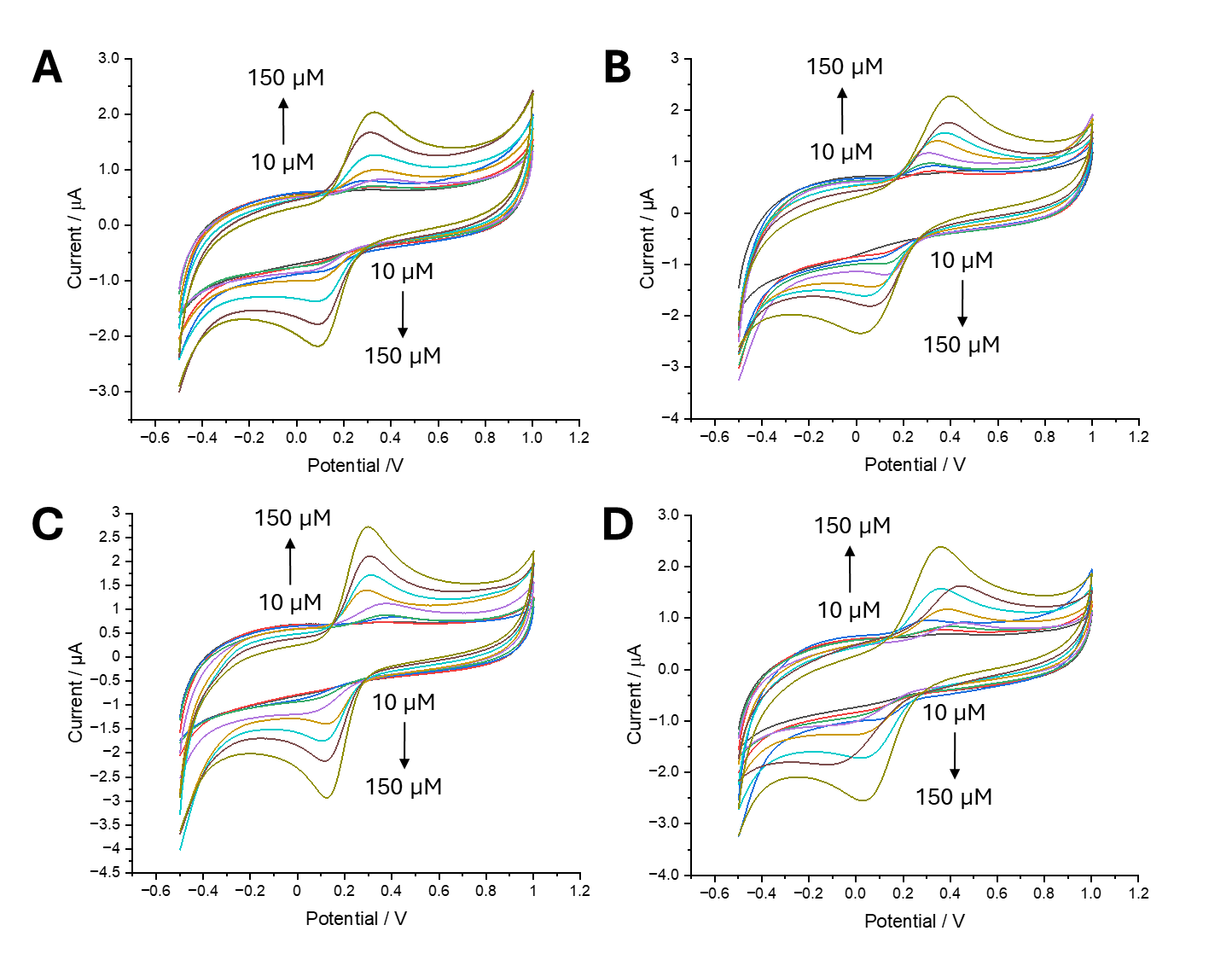


**Figure S1.** Series of cyclic voltammograms recorded in 250 mM NaCl for the increasing concentration of Fe(CN)_6_^3-/4-^ gelled with the following gelator: A – 2% gelatine; B – 0.5% agarose; C – 0.25% agarose; D – 0.25% agar-agar. The scan rate was 50 mV·s^-1^. Each CV was recorded in separate electrochemical cells.


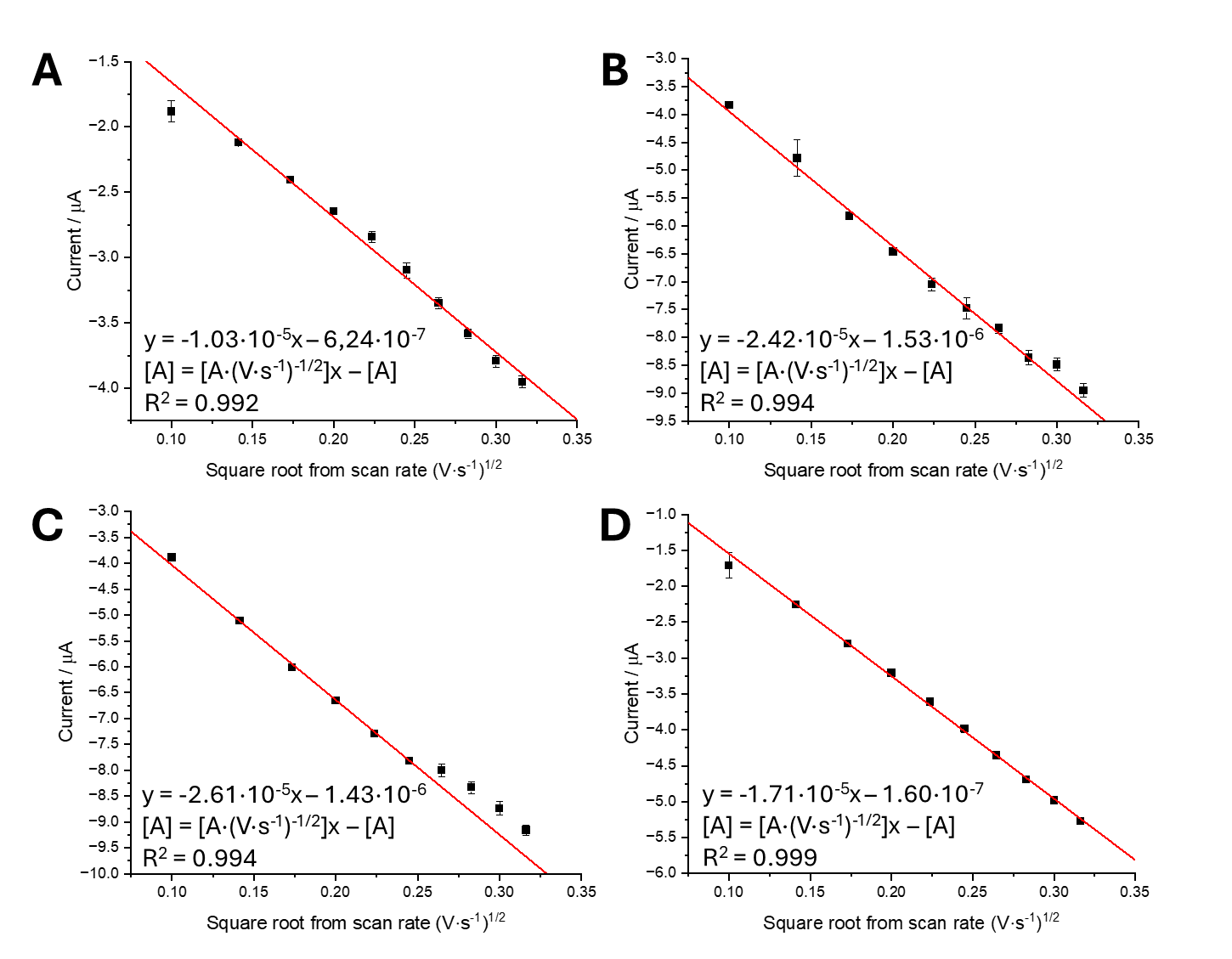


**Figure S2.** Current versus square root from scan rate dependencies recorded for fixed concentration of Fe(CN)_6_^3-/4-^ equal to 500 µM in 250 mM NaCl in the gelled electrolyte: A – 2% gelatine; B – 0.5% agarose; C – 0.25% agarose; D – 0,25% agar-agar. Scan rate values were in the range from 10 to 100 mV·s^-1^ with 10 mV·s^-1^ intervals.


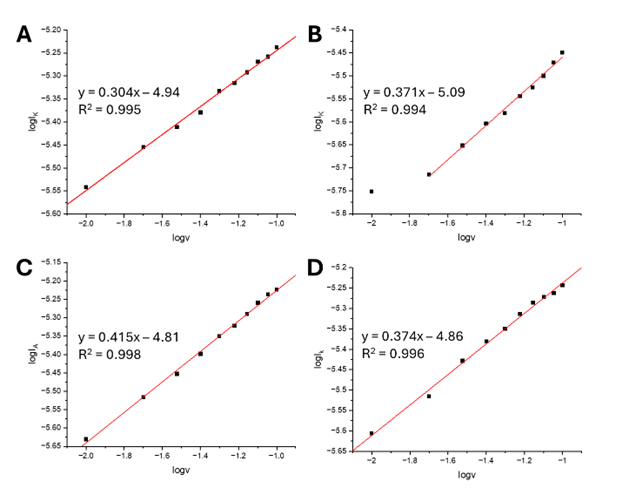


**Figure S3.** Series of logI_K_ versus logv dependencies recorded for fixed concentration of Fe(CN)_6_^3-/4-^ equal to 500 µM in 250 mM NaCl in the gelled electrolyte: A – 0% gelator (blank reading); B – 4% gelatine; C – 1.25% agarose; D – 0.5% agar-agar. Scan rate values were in the range from 10 to 100 mV·s^-1^ with 10 mV·s^-1^ intervals.


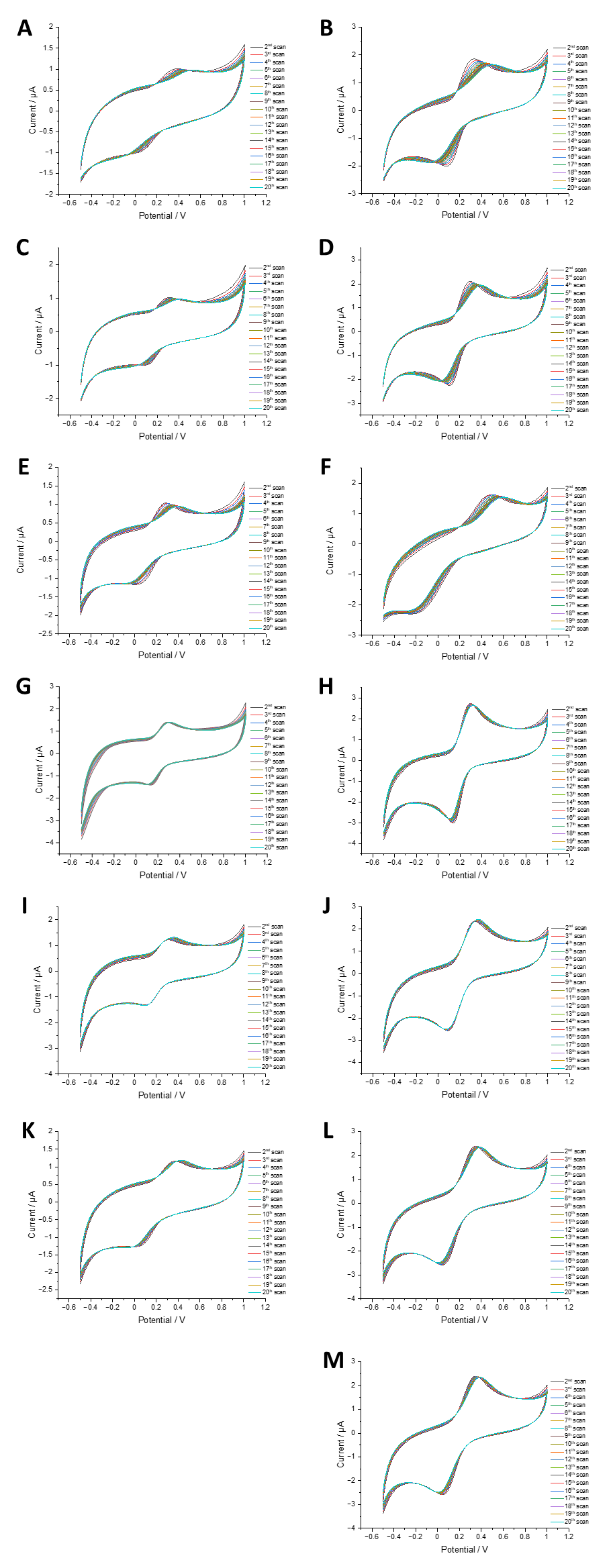


**Figure S4.** Series of cyclic voltammograms recorded for fixed concentration of Fe(CN)_6_^3-/4-^ equal to 50 µM (left panel) and 150 µM (right panel) in 250 mM NaCl in the gelled electrolyte: A, B – 4% gelatine; C, D – 2% gelatine ; E, F – 1.25% agarose; G, H – 0.25% agarose; I, J – 0.5% agar-agar; K, L – 0.25% agar-agar; M - 4% guar gum. The scan rate was 50 mV·s^-1^.
